# Recent advances in the genetic engineering of the *Leishmania* parasite and anti-cancer properties

**DOI:** 10.22038/ijbms.2025.87919.18992

**Published:** 2025

**Authors:** Saeid Rahim, Hossein Yousofi Darani, Hossein Khanahmad, Nadia Pourmoshir, Zahra Bakhshiyani, Sedigheh Saberi

**Affiliations:** 1 Department of Parasitology and Mycology, School of Medicine, Isfahan University of Medical Sciences, Isfahan, Iran; 2 Department of Genetics and Molecular Biology, School of Medicine, Isfahan University of Medical Sciences, Isfahan, Iran; 3 Department of Medical Biotechnology, School of Advanced Technologies in Medicine, Tehran University of Medical Sciences, Tehran, Iran; 4 Department of Medical Biotechnology, Faculty of Advanced Technologies, Shahrekord University of Medical Sciences, Shahrekord, Iran

**Keywords:** Attenuated, Host-pathogen interactions, Immunotherapy, Neoplasms, Protozoan infections, Reverse genetics, Vaccines

## Abstract

Leishmaniasis is a tropical disease caused by *Leishmania *species, affecting millions of people worldwide and contributing to substantial morbidity and mortality. Advances in genetic engineering technologies, particularly CRISPR/Cas9, plasmid shuffling, and DiCre-based systems, have significantly enhanced our understanding of *Leishmania *biology. These approaches have enabled precise gene editing, functional analysis of essential genes, and the development of genetically attenuated strains with potential applications in vaccine design and drug discovery. Gene editing tools have also allowed the identification of key virulence factors and pathways involved in parasite survival and modulation of the host immune system. These insights have opened new directions for therapeutic strategies against leishmaniasis. Interestingly, recent findings highlight notable similarities between leishmaniasis and cancer, including immune checkpoint involvement, chronic inflammation, and shared molecular targets. Leishmania’s ability to influence host immune responses and epigenetic mechanisms mirrors certain cancer-related processes. Moreover, compounds originally developed for cancer treatment, such as miltefosine and topoisomerase inhibitors, have shown effectiveness against *Leishmania*, supporting the potential for cross-applications. This review outlines recent developments in *Leishmania *genetic engineering and explores how these advancements can contribute to both anti-*leishmania*l and anti-cancer therapies. By emphasizing overlapping biological pathways and therapeutic targets, this work suggests innovative approaches to address two major global health challenges.

## Introduction

Leishmaniasis is a significant parasitic disease caused by over 21 species of the protozoan genus *Leishmania*, currently endemic in 99 countries and territories worldwide, according to the World Health Organization (WHO), posing a threat to more than 350 million individuals globally. Despite significant global health efforts, leishmaniasis remains a critical biomedical challenge due to limited therapeutic options, emerging drug resistance, and complex host–parasite interactions that are still poorly understood. These challenges highlight the importance of molecular approaches to understand parasite biology better and develop innovative interventions. Approximately 12 to 15 million individuals are currently infected, with 1.5 to 2 million new cases reported each year, resulting in an estimated 20,000 to 30,000 deaths annually (1). Leishmaniasis has been listed by the World Health Organization as one of the top 10 diseases common in tropical regions (2). *Leishmania* is transmitted through the bite of *Phlebotominae* sand flies (3). Leishmaniasis is a zoonotic disease that can manifest in three clinical forms: from asymptomatic infections to lesions of cutaneous leishmaniasis (CL), mucocutaneous leishmaniasis (MCL), or visceral leishmaniasis (VL) (4). CL is a dermal manifestation caused by various *Leishmania* species, such as *Leishmania tropica* and *Leishmania major* in the Eastern Hemisphere and *Leishmania* *mexicana*, *Leishmania* *viannia*, *Leishmania* *braziliensis*, *Leishmania guyanensis*, and *Leishmania panamensis* in the Western Hemisphere (5). CL has been documented in several countries neighboring Iran, such as Afghanistan, Iraq, Saudi Arabia, and Syria (6). Recent advancements in genetic engineering have revolutionized the understanding of *Leishmania* biology and facilitated the development of innovative therapeutic strategies, including vaccines. Among these, the use of genetically modified live attenuated vaccines stands out as a promising approach. Such vaccines involve the alteration or deletion of genes associated with the parasite’s virulence or intracellular survival, ensuring safety while eliciting robust immune responses (7).

Homologous recombination has been one of the primary methods used for generating null mutants in *Leishmania*, targeting virulence genes to create safer vaccine candidates. For instance, deletion of the dihydrofolate reductase-thymidylate synthase (DHFR-TS) gene was one of the earliest demonstrations of this approach, which significantly attenuated the virulence of *L. major* (8). Other targets include centrin, SIR2, and heat shock protein 70 (HSP70), all of which have shown promising immunogenicity and protective effects in animal models (9).

To overcome these limitations, several molecular tools have been developed to manipulate the *Leishmania* genome. Among the most widely used approaches are CRISPR/Cas9-based editing, plasmid shuffling, and DiCre-mediated recombination systems. These tools have enabled researchers to dissect gene function, evaluate virulence determinants, and construct live attenuated vaccine strains with greater precision and safety. Additionally, tools like CRISPR/Cas9 have enabled precise and efficient editing of the *Leishmania* genome, providing insights into gene functions and allowing the development of attenuated strains with enhanced safety profiles. Such techniques have the potential to refine vaccine candidates and improve their efficacy in inducing long-lasting immunity against visceral leishmaniasis (VL) (7).

Several studies have explored the role of *Leishmania* parasites in modulating the host immune system and their potential impacts on anti-cancer immunity. It has been reported that *Leishmania* parasites destabilize the host chromatin structure, which may lead to alterations in immune-related genes and responses (10-12).

A key similarity between leishmaniasis and cancer lies in the role of immune checkpoint molecules in regulating immune responses. Certain *Leishmania* species induce immune checkpoint molecules like Cytotoxic T-lymphocyte associated protein **4** (CTLA-4), which can suppress anti-parasitic immunity and contribute to disease progression (13, 14).

Additionally, parasite-derived molecules such as HSP100 play critical roles in parasite survival and interference with immune responses, resembling the function of similar molecules in the survival of cancer cells (15, 16). These parallels allow researchers to develop shared therapeutic approaches targeting both diseases.

Proteins such as HSP90 and HSP60, which are essential for cell stability and survival, are also significant in both leishmaniasis and cancer. Inhibitors of these proteins show potential not only in cancer therapy but also in managing leishmaniasis (17, 18).

Furthermore, certain chemical compounds like miltefosine, initially developed for cancer treatment, have demonstrated effectiveness in treating leishmaniasis. This suggests potential shared mechanisms in the pathological pathways of these two diseases (19, 20). 

This study aims to provide a comprehensive overview of recent advancements in the genetic engineering of *Leishmania*, with particular emphasis on their translational potential in the context of cancer therapy. By exploring overlapping molecular pathways, immune evasion strategies, and therapeutic targets, this review highlights a novel integrative perspective that bridges parasitology and oncology, suggesting that lessons learned from one field can directly benefit the other in the pursuit of more effective treatment strategies. However, despite these advances, significant challenges remain. The unique genomic architecture of *Leishmania*, characterized by polycistronic transcription and genome plasticity, complicates precise gene regulation and functional analyses. Furthermore, current gene-editing methodologies face limitations such as off-target effects and low transfection efficiencies, impeding large-scale functional genomics studies. Addressing these challenges is crucial for developing innovative therapeutic strategies and understanding the parasite’s interaction with host immune responses and potential implications in oncology.

### Genetic engineering of Leishmania

#### Molecular strategies for modifying Leishmania

More than 125 years have passed since *Leishmania* parasites were first identified as the etiologic agent of oriental cutaneous leishmaniasis by Piotr Fokich Borovsky in 1898. The first *Leishmania* genome was published in 2005, marking the beginning of a continuously expanding toolbox for the genetic manipulation of this vector-borne parasite(21). The challenges of conducting sexual crosses within the sandfly (22) and the presence of supernumerary chromosomes(23, 24) have made the analytical power of forward genetic methods to these diploid Organisms challenging. Given this scenario, the starting point for studying these infections is genome information, which is readily accessible through a variety of online resources such as TriTrypDB, LeishCyc, LeishBase, and TDR Targets. (25) combined with reverse genetics, -omics techniques, and bioinformatics has made it possible to investigate the study of non-essential genes involved in encoding proteins that facilitate infection, as well as the identification of essential genes that regulate critical cellular processes (26).

### Classic method of gene deletion by allelic substitution

It was shown in 1990 that *Leishmania* promastigotes could have their genes removed through homologous recombination using linear double-stranded DNA (dsDNA)(27). Cruz and colleagues provided proof of concept by deleting a solitary DHFR-TS allele in wild-type *L. major*. At the time, *Leishmania* was believed to possess a diploid genome, which allowed for the creation of null mutants from wild-type backgrounds. The development of novel resistance markers later enabled the deletion of both alleles of a gene. However, several studies indicated that the system would malfunction if both alleles of a putatively essential gene were deleted. Three possible outcomes were observed during drug selection: (1) no transfectants survived, (2) surviving parasites had duplicated their genome to retain both the drug resistance markers and the target gene (28), or (3) extra copies of the gene were found on extrachromosomal or ectopic elements. This was initially regarded as indirect evidence of gene essentiality. However, because this strategy could not be used to generate a true null mutant, it is now considered the least robust form of evidence, as the result might have been due to technical failure rather than biological necessity (29).

### Plasmid shuffling

The concept behind plasmid shuffle is that a plasmid containing an essential gene is only considered redundant if it is present in a functional copy or is accompanied by necessary metabolic products that compensate for its absence (30). Sensitivity to ganciclovir (GCV), a nucleoside analog, is conferred by the expression of an essential gene in conjunction with a herpes simplex thymidine kinase (TK) “suicide” cassette (31). The toxic metabolite GCV triphosphate, produced by active TK, prevents DNA synthesis and exerts significant selective pressure on the plasmid to be lost. A gene can be considered essential if parasites die or if, after negative selection, the plasmid is only required when another functional gene copy is present (32). An alternative method for positively identifying essential genes is plasmid shuffling. This approach requires determining whether the ectopic copy of a gene of interest is dispensable under negative selection, and then deleting the gene of interest from its genomic locus (33). 

**Figure 1:**
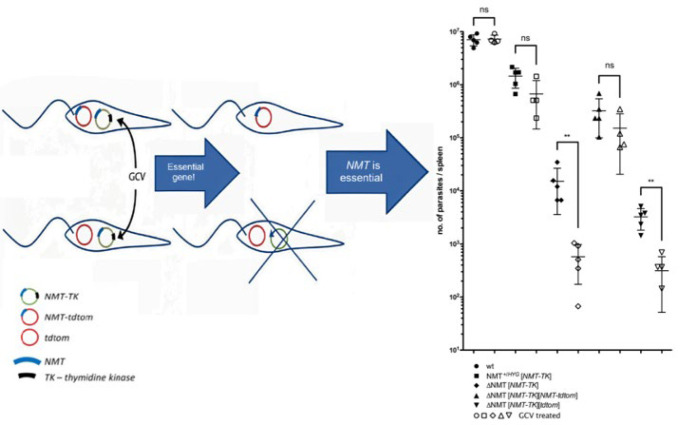
Plasmid shuffle strategy confirms NMT as an essential gene in *Leishmania*

The dual-function protein involved in the metabolism of 10-Formyl-THF, encoded by the *L. major* DHCH gene (31) and the *Leishmania* *donovani* CYP51 gene, retained on a TK expression plasmid during GCV treatment, is both critical for promastigote survival (34). Moreover, Daniel Paape and colleagues (2020) provided *in vivo* genetic evidence using plasmid shuffling techniques that N-myristoyltransferase (NMT) is essential for *Leishmania* viability at every stage of the parasite life cycle (Figure 1) (35). Since phenotype analysis of null mutants in the amastigote stage plays a vital role in validating drug targets, the plasmid shuffle technique must be compatible with this stage. However, a major limitation of the method is that null mutants of essential genes cannot be generated without GCV, a cytostatic rather than cytocidal drug, thereby preventing direct phenotype assessment of these mutants (36). The introduction of a second episome carrying mutant versions of the target gene represents an improvement in the method. This enables investigation of specific domains or residues by observing which plasmid is preferentially retained (37).

### Conditional transgenesis using Dimerizable Cre (DiCre)

**Figure 2:**
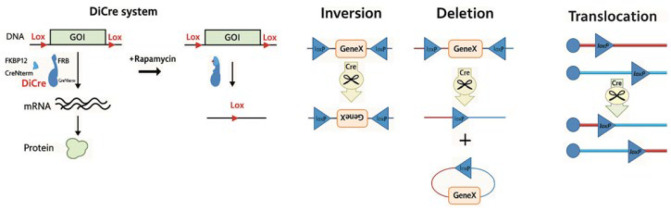
DiCre-based genetic engineering enables controlled gene inversion, deletion, and translocation

The DiCre-based inducible knockout expression system offers several advantages, including time- and dose-dependent control of gene expression and the ability to compare endogenous proteins with mutant versions. Compared to other inducible systems in *Leishmania*, DiCre requires fewer selectable markers, making it more straightforward to implement. Additionally, it is flexible and non-leaky, allowing gene expression to be induced from either chromosomal or episomal contexts (38). This site-specific recombinase (SSR) technology is based on the bacteriophage P1 Cre recombinase, a 38-kDa protein capable of catalyzing intra- and inter-molecular recombination between two loxP target sites. These loxP sequences are 34 base pairs (bp) long and consist of two 13-bp inverted repeats flanking an 8-bp asymmetrical core region. As this sequence is unique to the P1 phage genome and not naturally found in other genomes, the chance of random occurrence is extremely low. The orientation and position of the loxP sites determine whether deletion, insertion, or inversion of the DNA occurs (39). The system utilizes a single enzyme, Cre recombinase, divided into two enzymatically inactive polypeptides: an N-terminal CRE59 fragment and a C-terminal CRE60 fragment, each fused to distinct rapamycin-binding proteins (FKBP12 and FRB, respectively). Recombinase activity is reactivated when these two subunits heterodimerize in the presence of rapamycin, facilitating site-specific recombination (SSR) between the loxP sites, leading to the excision or inversion of the floxed target DNA segment (Figure 2a). This system enables precise DNA modifications, including deletions, insertions, translocations, and inversions at specific genomic sites (Figure 2-b) (38).

This powerful molecular biology tool, like other systems, has some limitations. First, Cre recombination efficiency varies depending on the cell type and construct. Second, Cre may cause recombination at cryptic or pseudo-loxP sites, estimated to occur in mammals at a frequency of 1.2 events per megabase. Additionally, Cre alone can sometimes generate phenotypes even in the absence of floxed constructs. This is particularly notable in *Drosophila* models (40).

### CRISPR/Cas9 gene editing revolution

**Figure 3:**
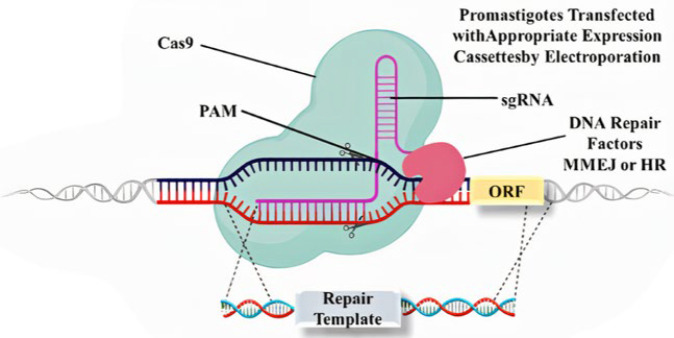
Mechanism of CRISPR-Cas9–based gene editing in *Leishmania* promastigotes

To analyze larger gene cohorts, high-throughput genetic methods are necessary for post-genomic investigations of parasite biology. The discovery of the CRISPR–Cas system, which is revolutionizing genome editing across eukaryotes (41), including protozoan parasites (42, 43), came about as an RNA-mediated adaptive defensive mechanism in bacteria and archaea (44). The CRISPR/Cas9 method was developed using an RNA-guided endonuclease CRISPR-associated (Cas) protein’s capacity to precisely create double-strand breaks and silence foreign DNA (45). The single guide RNA (sgRNA) and the endonuclease Cas9 are the two essential parts of this technology. With the assistance of a certain sgRNA, the Cas9 (which is usually generated from Streptococcus pyogenes) can produce a double-strand break at the target locus. By Watson–Crick pairing of 20 nucleotides with the genomic DNA, the sgRNA can “guide” the endonuclease Cas9 to the target region (Figure 3) (46). Using a range of promoters, such as ribosomal RNA promoters (47), U6 (32), or T7 (48), sgRNA molecules can be produced (49) or transcribed from template DNA (50). Consequently, sgRNA and Cas9 combine to create a ribonucleoprotein complex that can localize the sgRNA’s complementary genomic sequence. Only when a protospacer-adjacent motif (PAM) is present on both sides of the 20 nucleotides complementary to the sequence can this complex develop, and a double-strand break is generated (46). The CRISPR/Cas9 approach, therefore, offers a customizable endonuclease that can target and/or split a sequence of DNA by providing an intentionally constructed guide RNA (51). To produce the single-guide RNA (sgRNA), it is sufficient to append a 20-nucleotide sequence complementary to the target DNA at the 5′ end of a universal Cas9-binding gRNA scaffold (52). This cassette contains homology regions to facilitate homology-directed repair, along with a selectable marker to identify and isolate the mutant. The specific design of the cassette depends on the intended genetic modification. Repair of the subsequent double-strand break (DSB) happens in kinetoplastids by a mechanism known as microhomology-mediated end joining (MMEJ) (47) rather than nonhomologous end joining (NHEJ), which is the predominant pathway in mammalian cells. MMEJ depends on short stretches of sequence identity on either side of the DSB. This repair mechanism enables precise gene editing by providing repair templates with homology flanks, which can be as short as 24 nucleotides (48).

Shortly after the system’s adoption in 2014, the first CRISPR/Cas9 report in Kinetoplastida was published, focusing on Trypanosoma cruzi (50). Following the system’s installation in *Leishmania*, report mechanisms were implemented in *L. major *(53) and *Leishmania donovani* (47). The subsequent findings focused on plasmid-based approaches that used vectors to express Cas9 and sgRNA. For every target gene of interest, cloning procedures must be used to modify the elements, sgRNA, and donor DNA. A CRISPR/Cas9 strategy based on PCR and without cloning was published by Beneke *et al*. (48) and greatly enhanced these earlier techniques.

### Potential anti-cancer applications of Leishmania

One of the most promising areas of *Leishmania* research involves its potential applications in cancer therapy. The parasite’s unique biology and capacity to modulate host immune responses position it as a potential candidate for novel anti-cancer strategies.

### Genetically engineered Leishmania for tumor targeting

*Leishmania* possesses several features that make it a valuable platform for cancer therapy. These include its tropism for macrophages, the ability to activate host immune responses, and ease of genetic manipulation. Genetically engineered strains can be designed to express therapeutic proteins, tumor-associated antigens (TAAs), or cytokines that modulate tumor microenvironments.

For instance, *Leishmania* tarentolae, a non-pathogenic species, has been used as a vehicle to deliver antigens to dendritic cells (DCs), enhancing T-cell responses against tumors (54). The ability to engineer *Leishmania* to express IL-12 or GM-CSF further underscores its potential as a cancer immunotherapy tool (55).

Moreover, attenuated *Leishmania* strains can serve as live vaccines or delivery vectors. A notable example includes the LdCen^−/− strain, which showed strong activation of cytotoxic T lymphocytes (CTLs) and inhibition of tumor growth in murine models (56).

### Oncolytic Leishmania

Like oncolytic viruses, genetically modified *Leishmania* can be programmed to selectively replicate in tumor cells or modulate tumor-associated immune cells. Although *Leishmania* is not naturally cytolytic, its ability to persist intracellularly and interact with antigen-presenting cells makes it an ideal platform for oncolytic strategies.

Additionally, *Leishmania* components such as lipophosphoglycan (LPG) and glycosylphosphatidylinositol (GPI) anchors possess inherent adjuvant properties that may enhance anti-tumor immunity. When combined with tumor-specific antigens, these molecules can potentiate robust immune responses (57).

### Immunomodulation by Leishmania

One of the most powerful mechanisms by which *Leishmania* may exert anti-cancer effects is through immune modulation. The parasite can influence both innate and adaptive immune responses, shifting the balance from a tumor-permissive (Th2) to a tumor-rejecting (Th1) profile.

Live *Leishmania* parasites have been shown to stimulate macrophages, DCs, and natural killer (NK) cells, promoting the release of pro-inflammatory cytokines such as IFN-γ and IL-12. These cytokines are critical for initiating anti-tumor immunity (58).

Furthermore, *Leishmania*-infected macrophages exhibit enhanced antigen presentation and costimulatory molecule expression, leading to improved T cell priming. This activation can break tumor-induced tolerance and restore effective immune surveillance.

### Leishmania-derived molecules with anti-cancer properties

In addition to whole parasites, several *Leishmania*-derived molecules have demonstrated potential as anti-cancer agents. For example, KMP-11 (kinetoplastid membrane protein-11), a conserved protein across kinetoplastids, exhibits apoptotic activity against tumor cells (59).

Studies have also identified parasite lipids and glycoproteins capable of modulating host cell signaling pathways, inducing apoptosis, or inhibiting angiogenesis in cancer models. LPG, in particular, can downregulate tumor-promoting cytokines and interfere with metastatic processes (60).

These findings open up avenues for using *Leishmania*-derived molecules as templates for designing novel anti-cancer drugs, immunotherapeutics, or vaccine adjuvants.

### Candidate genes and pathways for therapy development

A deeper understanding of the molecular biology of *Leishmania* has uncovered several genes and pathways that are shared with cancer cells. These targets are pivotal for survival, pathogenicity, and immune modulation in both diseases:

### Topoisomerases (TOPs)

Topoisomerases play crucial roles in DNA replication, transcription, and repair, making them indispensable for cellular proliferation. In *Leishmania*, type IB and type II topoisomerases are particularly important for the organization and replication of kinetoplast DNA. Camptothecin derivatives, commonly used as anti-cancer agents, have demonstrated promising leishmanicidal activity by inhibiting these enzymes (61, 62). 

### Heat shock proteins (HSPs)

HSPs such as HSP60 and HSP90 are molecular chaperones that stabilize client proteins, which facilitates cellular adaptation to stress. Overexpression of HSPs has been linked to drug resistance in both cancer and *Leishmania*. Specific inhibitors like geldanamycin and its derivatives (e.g., 17-AAG) have been effective in disrupting HSP90 function in both diseases. For *Leishmania*, these inhibitors impair survival by targeting the N-terminal domain of HSP90. This domain is crucial for parasite adaptation within the host (18, 63).

### Phosphoglycerate kinase-1 (PGK-1) 

PGK-1 is an enzyme involved in glycolysis and energy metabolism, and it plays dual roles depending on the cellular environment. In cancer, PGK-1 contributes to angiogenesis inhibition under extracellular conditions, whereas it promotes ATP production under hypoxic conditions. In *Leishmania*, PGK-1 is associated with antimony resistance and energy metabolism, highlighting its role in parasite survival. Targeting this enzyme with small-molecule inhibitors could represent a promising therapeutic strategy for both diseases (64, 65).

### Tubulins

Tubulin proteins, which are critical for cytoskeletal integrity and cell division, are conserved across eukaryotic organisms. In *Leishmania*, tubulins contribute to flagellar motility and drug resistance. Tubulin inhibitors, such as colchicine derivatives and paclitaxel analogs, are widely used in cancer therapy and have shown potential as treatments for leishmaniasis (66, 67).

### Selenoproteins

Selenoproteins, known for their anti-oxidant properties, are involved in managing oxidative stress in both cancer and *Leishmania*. Selenocompounds have shown leishmanicidal activity by inhibiting selenoproteins, which are essential for parasite survival. They also disrupt redox homeostasis in cancer cells, potentially enhancing therapeutic effects (68, 69).

### GP63

GP63 facilitates the binding of *Leishmania* to macrophages by interacting with fibronectin receptors on the macrophage surface. In contrast, the primary surface molecules of metacyclic promastigotes, such as lipophosphoglycans, do not play a role in the macrophage uptake process. *Leishmania* lacking GP63 are less capable of infecting macrophages compared to their wild-type counterparts. In general, GP63 is essential for interaction with host cells and promotes *Leishmania* survival within the phagosomes (70).

### Surface lipophosphoglycans

Lipophosphoglycan (LPG), a glycophosphatidylinositol (GPI)-anchored molecule, dominates the surface of promastigotes. Its structural composition and sugar sequence vary across species. By interacting with lectins on the sandfly’s gut lining, LPG plays a vital role in enabling the parasite to establish infection in its vector (71).

### KMP-11 (kinetoplastid membrane protein-11)

An LPG-related protein that is highly antigenic for mouse and human T cells. This protein is 11-kDa and is present in various kinetoplastids. It is expressed during the life cycle of *Leishmania*, with increased expression in the metacyclic and amastigote stages, which indicates an important role in the mammalian host, such as reducing iNOS activity in infected macrophages (72). KMP-11 protein is involved in binding to the host cell (73).

### Cysteine proteinases

Cysteine proteinases (CPs) are classified as common virulence factors in the *L.* *mexicana* species, whose inhibition can be related to the control of this infection. CPs are enzymes recognized for their crucial involvement in the pathogenesis of infections caused by various parasitic protozoa (74, 75). The most extensively studied cysteine proteases, CPs, in *Leishmania* are CPA, CPB, and CPC, all of which belong to the papain-like family. Numerous CP genes have been identified and characterized in *Leishmania*, particularly within species of the *L.* *mexicana *complex, including *L.* *mexicana*, *Leishmania* *pifanoi*, and *Leishmania* *amazonensis* (76-79). The genomic structure and characteristics of the cathepsin L-like cysteine proteinase gene cluster in the *Leishmania* *donovani* complex have been previously documented. Additionally, single-nucleotide polymorphisms (SNPs) have been identified in CPs, with variations observed depending on the life stage of the parasite. CPs play a vital role in the basic functions and interactions of *Leishmania* *tropica* with the host (80).

### P46

The gene, P46 (LmjF33.0360), encodes a 46 kDa protein with virulence properties for the parasite. Overexpression of this protein rescues the virulence of Hsp100 null mutants and enhances lesion formation in the *L. major* BALB/c mouse strain (81). P46 can be part of immunomodulatory exosomes of *L. major* that accumulate in the cytoplasm of infected macrophages. Finally, P46 improves the ability of parasites to survive intracellularly (82).

### Centrin

One of the candidates for vaccine production is the centrin protein. This protein binds to calcium in *Leishmania* and is located in the basal body of the parasite, where it regulates centrosome proliferation. It is coded by five genes in *Leishmania*. *Leishmania* centrin 1-3 is similar to human centrin 1-3 in that it has two possible binding sites for calcium. Centrin4 and centrin5 exclusively belong to the Trypanosomatidae family (83). One of the most important of them is the *Leishmania* centrin-1 gene, which is effective in the reproduction, growth, and differentiation of promastigotes to amastigotes. Deletion of centrin in *Leishmania* leads to a decrease in IL-10 and an increase in the production of IFN-γ and TNF-α by CD4+ T cells, ultimately causing strong innate immune responses that support T cell anti-parasitic activity (84, 85). 

### Trypanothione reductase (TR)

There are different mechanisms for *Leishmania* to survive in the macrophage; one of them is the activation of the enzyme trypanothione reductase, which neutralizes reactive oxygen species produced inside the macrophages, allowing the parasite to survive within the macrophage (86). Trypanothione reductase belongs to the family of disulfide oxidoreductase enzymes, which are unique to parasites and absent in humans. This enzyme performs oxidoreductive reactions in the human body through an analogue called glutathione reductase (GR) (87). One of the most important drugs in the treatment of leishmaniasis is antimonials, which inhibit the parasite’s metabolism by interfering with trypanothione (88). One of the reasons that makes TR a suitable research target is its low toxicity, high level of genetic validity, and detailed structural information (89). However, the competitive TR inhibitors developed so far have shown low potency (90).

## Discussion

Recent advancements in *Leishmania* genetic engineering techniques have provided groundbreaking insights into the biology of this parasite and its potential applications in both therapeutic and vaccine development. Key innovations such as CRISPR/Cas9 gene editing, plasmid shuffling, and dimerizable Cre (DiCre) systems have enabled precise and efficient modifications of the *Leishmania* genome, facilitating the identification of essential genes, virulence factors, and novel drug targets. These tools have significantly improved our ability to create live attenuated vaccine candidates, which can elicit robust and long-lasting immune responses while ensuring safety profiles suitable for clinical use (91-93)

The integration of reverse genetics and omics-based approaches has further revealed critical insights into the parasite’s survival mechanisms and host interactions. For instance, targeting genes such as centrin, DHFR-TS, and NMT has not only validated their essential roles but also demonstrated their potential as drug targets (94). These techniques offer a new paradigm in combating leishmaniasis by combining therapeutic interventions with advanced molecular tools.

Additionally, the shared molecular and immunological mechanisms between leishmaniasis and cancer, such as the role of immune checkpoints (e.g., CTLA-4, PD-L1) and pro-inflammatory pathways mediated by TLR signaling, open up novel avenues for therapeutic development (95). This overlap highlights the possibility of leveraging knowledge from cancer research to design innovative therapies for leishmaniasis and vice versa.

Studies reveal that *Leishmania* and cancer share common biological pathways that influence disease progression and host immune responses. A key aspect is the role of chronic inflammation in creating a microenvironment conducive to carcinogenesis. *Leishmania* infections disrupt the function of immune cells such as macrophages and dendritic cells, fostering a pro-inflammatory environment enriched with Th2 responses, which resembles the tumor microenvironment. Additionally, scars resulting from CL have been proposed as potential sites for precancerous or cancerous changes. This phenomenon has been particularly observed in cases of basal cell carcinoma (BCC) and squamous cell carcinoma (SCC) developing in *Leishmania*-affected scar tissues. Research into therapeutic applications of *Leishmania* has further strengthened this connection. Compounds originally developed for cancer treatment have shown efficacy against *Leishmania*. For example, miltefosine, first designed as an anticancer agent, is now the first oral drug approved for leishmaniasis treatment, underscoring shared mechanistic pathways between the two diseases (96). Similarly, topoisomerase I inhibitors, such as camptothecin, widely used in cancer therapy, have demonstrated activity against *Leishmania donovani* topoisomerase, highlighting the therapeutic overlap and potential for drug repurposing (97).

This study’s strength lies in its integrated approach, which bridges molecular parasitology with cancer immunology and leverages recent advances in genome editing. The inclusion of up-to-date insights and translational relevance further reinforces its scientific value. However, certain limitations should be acknowledged. Many findings are based on laboratory-adapted *Leishmania* strains and murine models, which may not fully replicate human immune dynamics or genetic diversity. These differences limit the direct translatability of results. Additionally, more *in vivo *studies in endemic regions and validation using clinical isolates are needed to enhance relevance and generalizability.

NMT: N-myristoyltransferase

**Table 1:** Overview of widely used genetic engineering strategies in *Leishmania* species, highlighting their key advantages, disadvantages, and typical applications in functional genomics and gene manipulation studies

Method	Advantages	Disadvantages	Applications	Ref.
CRISPR/Cas9	High precision enables multigene editing, efficient knockout of multi-copy genes	Requires Cas9/gRNA expression, off-target risks, and low transfection efficiency	Gene knockout, tagging, point mutations, functional genomics	(92)
DiCre/loxP system	Conditional deletion of essential genes, temporal regulation	Requires dual loxP insertion, labor-intensive setup	Essential gene analysis, conditional mutants	(98)
LeishGEdit toolkit	No cloning, high efficiency, compatible with large-scale screens	Requires stable Cas9 and T7 polymerase expression	Gene knockout, tagging, and high-throughput functional screening	(99)
Base Editing (Cas12a)	No double-strand breaks, ideal for precise point mutations	Lower efficiency in some species requires accurate crRNA design	Precise point mutation studies and regulatory analysis	(100)
RNA interference (RNAi)	Transient silencing, no permanent genome alterations	Limited to *Viannia* species, ineffective in *L. major*	Gene expression regulation, loss-of-function studies	(101)
Plasmid Shuffling	Suitable for complementation and essential gene validation	Time-consuming, requires complex plasmid design	Essentiality testing, gene rescue assays	(27)

## Conclusion

Recent advances in genetic engineering of *Leishmania* have significantly deepened our understanding of the parasite’s biology and unveiled novel avenues for the development of safer and more effective therapeutic and prophylactic interventions. Cutting-edge technologies, such as CRISPR/Cas9 and plasmid shuffling, enable precise targeting of essential molecular pathways, thereby offering promising strategies to combat this complex and debilitating disease.

Furthermore, the molecular and immunological parallels between leishmaniasis and cancer present a compelling framework for cross-disciplinary therapeutic innovation. The identification of shared targets, including heat shock proteins (HSPs), GP63, and topoisomerases, highlights the potential to repurpose anticancer agents for antileishmanial applications, fostering a synergistic approach to treatment development.

Moving forward, continued integration of advanced genetic tools with immunological and biochemical insights will be pivotal in refining these strategies. Rigorous preclinical evaluation and well-designed clinical trials are imperative to translate these findings into safe and effective therapies. Ultimately, sustained collaborative research efforts and technological advancements hold the promise to transform the therapeutic landscape of leishmaniasis and contribute meaningfully to oncology, underscoring the value of interdisciplinary approaches in tackling complex diseases.

## Declaration

To prepare for this study, the authors used ChatGPT to reduce plagiarism and improve language and grammar. The authors then re-reviewed and edited the article.
